# Multidisciplinary Medical Emergency Team System increases survival in cancer patients undergoing cardiopulmonary resuscitation

**DOI:** 10.1186/cc12236

**Published:** 2013-03-19

**Authors:** L Hajjar, F Galas, S Vieira, J Almeida, E Osawa, C Park, J Fukushima, E Angelo, A Pinheiro, J Auler Jr

**Affiliations:** 1 Heart Institute, São Paulo, Brazil; 2 Cancer Institute of University of São Paulo, Brazil

## Introduction

In the last years, ICU admission has been high in cancer patients due to increased survival related to advances in treatment. Patients with cancer reportedly have poor outcomes from cardiopulmonary resuscitation (CPR). The goal of this study was to evaluate the effectiveness of a multidisciplinary Medical Emergency Team System in the CPR outcomes in cancer patients.

## Methods

We performed a prospective study between January 2011 and December 2012 at a university cancer reference hospital in Brazil. Consecutive patients undergoing cardiac arrest and receiving CPR were identified, and a database was used to register baseline characteristics of patients, data for CPR and 30-day survival. During the first 12 months of the study (phase 1), patients received CPR according to the institution's protocol guided by different physicians. During the last 11 months of the study (phase 2), a multidisciplinary Medical Emergency Team System consisting of a specialized team of ICU nurses, physiotherapists and physicians using an electronic calling system was responsible for the CPR.

## Results

A total of 182 patients experienced in-hospital cardiac arrest and received CPR. Most patients were in hospital due to medical admission (82%), had solid tumors (85%), and had localized disease (60%). Pulseless electric activity was the predominant arrest rhythm (54%) and 45% of patients were resuscitated on the ICU. Overall 30-day survival was 9.3%. In the phase 1 of the study, 30-day survival was 3.7%, and in phase 2, survival was 14% (*P *= 0.017). There were no differences in the two phases of the study regarding baseline characteristics of patients, rhythm and place of CPR.

## Conclusion

Overall survival from CPR in cancer patients compares favorably with survival rates in noncancer patients. A multidisciplinary Medical Emergency Team System increases survival in cancer patients undergoing cardiopulmonary resuscitation.
